# Reduction or de-escalation of dual antiplatelet therapy intensity or duration in patients with acute coronary syndromes undergoing percutaneous coronary intervention: A mini-review

**DOI:** 10.3389/fcvm.2022.1018649

**Published:** 2022-10-20

**Authors:** Mohamed Farag, Visvesh Jeyalan, Jose Luis Ferreiro, Young-Hoon Jeong, Tobias Geisler, Diana A. Gorog

**Affiliations:** ^1^Department of Cardiology, East and North Hertfordshire NHS Trust, Stevenage, United Kingdom; ^2^Department of Clinical, Pharmaceutical and Biological Science, School of Life and Medical Sciences, University of Hertfordshire, Hatfield, United Kingdom; ^3^Department of Cardiothoracic, Freeman Hospital, Newcastle upon Tyne, United Kingdom; ^4^Department of Cardiology, CIBERCV, Hospital Universitario de Bellvitge, L'Hospitalet de Llobregat, Spain; ^5^Bio-Heart Cardiovascular Diseases Research Group, Bellvitge Biomedical Research Institute (IDIBELL), L'Hospitalet de Llobregat, Spain; ^6^CAU Thrombosis and Biomarker Center, Chung-Ang University Gwangmyeong Hospital, Gwangmyeong-si, South Korea; ^7^Department of Internal Medicine, Chung-Ang University College of Medicine, Seoul, South Korea; ^8^Department of Cardiology and Angiology, University Hospital, Eberhard-Karls-University Tuebingen, Tübingen, Germany; ^9^Imperial College, National Heart and Lung Institute, London, United Kingdom

**Keywords:** acute coronary syndrome, PCI, antiplatelet therapy, P2Y_12_ inhibitor, de-escalation

## Abstract

Current guidelines for patients with acute coronary syndrome (ACS) recommend dual antiplatelet therapy (DAPT) for 12 months. Since bleeding is the main Achilles' heel of DAPT, in recent years several randomized controlled trials have evaluated the safety and efficacy of de-escalation of DAPT with respect to ischaemic and bleeding endpoints. These trials can be broadly divided into studies evaluating a shorter duration of DAPT, and those studies in which DAPT that includes a potent P2Y_12_ inhibitor, such as prasugrel or ticagrelor, is compared to less intense DAPT, mainly clopidogrel or reduced-dose prasugrel. We sought to evaluate the studies assessing de-escalation of DAPT in patients with ACS undergoing PCI. We review the studies evaluating the strategies of de-escalation of DAPT intensity and those evaluating a strategy of de-escalation of DAPT duration in ACS patients undergoing PCI. We summarize the limitations of studies to date, gaps in evidence and make recommendations for future studies.

## Introduction

Dual antiplatelet therapy (DAPT) is the cornerstone of treatment for patients with acute coronary syndromes (ACS) undergoing percutaneous coronary intervention (PCI). Current ESC guidelines recommend 1 year of DAPT unless contraindicated or if the bleeding risk is excessive (1–3). These guidelines also recommend use of a potent P2Y_12_ inhibitor, namely ticagrelor or prasugrel, over clopidogrel. However, this duration and intensity of DAPT exposes patients to increased bleeding risk, which is emerging as at least an equal, if not greater concern, than the ischaemic risk, with significant impact on mortality (4–6). Increased awareness of the prognostic importance of bleeding, together with observed increase in bleeding rates have prompted studies that consider alternatives to 12 months of high-intensity DAPT to balance thrombotic and bleeding risks. Several randomized controlled trials have investigated various de-escalation strategies in ACS patients undergoing PCI, either by reducing the intensity of DAPT, through switching from more potent P2Y_12_ inhibitors prasugrel or ticagrelor to clopidogrel, or by shortening the duration of DAPT and continuing with single antiplatelet therapy (SAPT). We sought to review the evidence supporting de-escalation of DAPT in patients with ACS undergoing PCI.

## Landmark trials establishing standard of care

The TRITON-TIMI 38 and PLATO multicentre randomized controlled trials were the first to compare the effectiveness of DAPT containing prasugrel or ticagrelor, with DAPT containing clopidogrel, in ACS patients including those undergoing PCI (7–9). The TRITON-TIMI 38 trial compared prasugrel to clopidogrel, in combination with aspirin, and all patients underwent revascularization (7, 8). The PLATO trial compared 12 months of ticagrelor to clopidogrel, in combination with aspirin (9), with 65% of patients undergoing revascularisation. Both trials demonstrated a reduction in ischaemic events within the first 30 days, whereas the difference in bleeding was mainly seen after this period. These trials led to the preferential recommendation in the ESC Guidelines for prasugrel or ticagrelor over clopidogrel in ACS patients undergoing PCI (1–3). Notably, in PLATO and TRITON-TIMI 38, few patients were aged ≥75 years (15 and 13%, respectively), a fewer than seen amongst ACS patients in daily practice, although the benefit of ticagrelor was seen regardless of age, in PLATO (9), but not in TRITON-TIMI 38 (7).

## Trials assessing de-escalation strategies

Twenty-five prospective trials assessed de-escalation of DAPT duration or intensity in ACS (Tables 1, 2). We excluded those studies in which ACS patients formed only a minority of the cohort, or when randomization occurred beyond 3 months after post-ACS (36–38). We present trial data including the trial-defined primary efficacy endpoint, which most often included major adverse cardiovascular events (MACE), namely the composite of death, myocardial infarction (MI) and stroke or net adverse cardiovascular events (NACE, composite of MACE and trial-defined bleeding) and the primary safety endpoint of bleeding (major or clinically-relevant non-major bleeding).

**Table 1:** Reduced intensity or de-escalation of dual antiplatelet therapy intensity in ACS population undergoing PCI.

**Study, year**	**Study design**	**DAPT strategy**	**Population (*n*)**	**Follow up (months)**	**ACS (%)**	**PCI (%)**	**Results**
PRASFIT ACS, 2014 (10)	Randomized Double-blinded Multicentre	**Intervention arm:** Aspirin (81–100) mg od and Prasugrel 3.75 mg od	685; East Asian population	6	UA: 20.5%; NSTEMI: 29.3%; STEMI: 50%	100%	**Efficacy endpoint** Composite of CV death, nonfatal MI, and ischaemic stroke: 9.4% in intervention group vs. 11.8% in control group (RR 23%; HR 0.77, 95% CI 0.56–1.07) **Safety endpoint** Non-CABG related TIMI major bleeding: 1.9% in intervention group vs. 2.2% in control group (HR 0.82; 95% CI 0.39–1.73)
		**Control arm** Aspirin (81–100) mg od and Clopidogrel 75 mg od	678; East Asian population				
PHILO, 2015 (11)	Randomized Double-blinded Multicentre	**Intervention arm:** Aspirin (75–100) mg od and Ticagrelor 90 mg bd	401; East Asian population	12	UA: 28.4%; NSTEMI: 17.5%; STEMI: 51.8%	84.6%	**Efficacy endpoint** Composite of MI, stroke, or death from vascular causes: 9.0% in intervention group vs. 6.3.% in control group (HR 1.47; 95% CI 0.88–2.44) **Safety endpoint** First occurrence of any major bleeding event according to PLATO criteria: 10.3% in intervention group vs. 6.8% in control group (HR 1.54; 95% CI 0.94–2.53)
		**Control arm:** Aspirin (75–100) mg od and Clopidogrel 75 mg od	400; East Asian population				
Tang et al. (12)	Randomized Double-Blinded Multicentre	**Intervention arm:** Aspirin 100 mg od and Ticagrelor 90 mg bd	200: East Asian population	6	STEMI: 100%	100%	**Efficacy endpoints** Composite of overall death, MI, unplanned revascularization, and stroke: 5% in intervention group vs. 14% in control group (OR 0.341; 95% CI 0.120–0.964; *P =* 0.034) Composite of CV death, nonfatal MI, and stroke: 4% in intervention group vs. 13% in control group (OR 0.294; 95% CI 0.09–0.916; *P =* 0.026) **Safety endpoint** Composite endpoint of major and minor TIMI bleeding: 10% in intervention group vs. 7% in control group (OR 1.451; 95% CI 0.541–3.891; *P =* 0.457)
		**Control arm:** Aspirin 100 mg od and Clopidogrel 75 mg od	200: East Asian population				
Wang et al. (13)	Randomized Double-blinded Single center	**Intervention arm:** Aspirin 100 mg od and Ticagrelor 90 mg bd	100: East Asian population	12	UA: 20%; NSTEMI: 45.5%; STEMI: 34.5%	73.5%	**Efficacy endpoint** Composite of CV death, MI, and stroke: 11% in intervention group vs. 22% in control group (HR 0.473; 95% CI 0.230–0.976; *P =* 0.043) **Safety endpoint** PLATO major bleeding: 8% in intervention group vs. 6% in control group (HR 1.250 :95% CI 0.434–3.604; *P =* 0.679)
		**Control arm:** Aspirin 100 mg od and Clopidogrel 75 mg od	100: East Asian population				
TROPICAL ACS, 2017 (14)	Randomized Open label Multicentre Platelet function guided de-escalation	**Intervention arm:** Aspirin 100 mg od and Prasugrel 10 mg or 5 mg od (based on age and weight) for 1 week, then Clopidogrel 75 mg od for 1 week, then platelet function testing. If high platelet reactivity documented, then switched back to Prasugrel, otherwise Clopidogrel for 1 year	1,304	12	NSTEMI: 45%; STEMI: 55%	100%	**Primary endpoints** Composite of CV death, MI, stroke, or BARC ≥2 bleeding: 7% in intervention group vs. 9% in control group (HR 0·81; 95% CI 0.62–1.06; P for noninferiority =0.0004; P for superiority =0.12) No significant difference in ischaemic endpoints. **Safety endpoint** Bleeding [BARC] ≥2: 5% in intervention group vs. 6% in control group (HR 0·82; 95% CI 0.59–1.13; *P =* 0·23)
		**Control arm**: Aspirin 100 mg od and Prasugrel 10 mg or 5 mg od (based on age and weight) for 1 year	1306				
TOPIC, 2017 (15)	Randomized Open label Single center	**Intervention arm**: Aspirin and a potent P2Y_12_ inhibitor (Ticagrelor/ Prasugrel) for 1 month, then switched to Aspirin 75 mg od and Clopidogrel 75 mg od for 11 months thereafter	322	12	UA/NSTEMI: 60%; STEMI: 40%	100%	**Primary endpoints** Composite of CV death, urgent revascularization, stroke, or BARC ≥2 bleeding: 13.4% in intervention group vs. 26.3% in control group (HR 0.48; 95% CI 0.34–0.68; P < 0.01). No significant difference in ischaemic endpoints. **Safety endpoint** BARC bleeding ≥2: 4.0% in intervention group vs. 14.9% in control group (HR 0.30; 95% CI 0.18–0.50; P < 0.01)
		**Control arm**: Aspirin and a potent P2Y_12_ inhibitor (Ticagrelor/ Prasugrel) for 12 months	323				
Elderly ACS-2, 2018 (16)	Randomized Open label Multicentre	**Intervention arm**: Aspirin 75–100 mg od and Prasugrel 5 mg od for 12 months	713: Elderly population	12	UA: 10%; NSTEMI: 48%; STEMI: 42%	99.5%	**Primary endpoints** Composite of all-cause death, MI, stroke, CV rehospitalization or bleeding [BARC 2-3]: 17.0% in intervention group vs. 16.6%, in control group (HR 1.007; 95% CI 0.78–1.30; *P =* 0.955) No significant difference in ischaemic endpoints. **Safety endpoint** BARC bleeding ≥2: 4.1% in intervention group vs. 2.7 % in control group (HR 1.52; 95% CI 0.85–3.16; *P =* 0.18)
		**Control arm**: Aspirin 75–100 mg od and Clopidogrel 75 mg od for 12 months	730: Elderly population				
PRAGUE-18, 2018 (17)	Randomized Open label Multicentre	**Intervention arm**: Aspirin 100 mg od and Prasugrel 10 mg od, or 5 mg od (based on age and weight)	634	12	STEMI; 89.5%; High-risk NSTEMI; 5.5%	99.2%	**Efficacy endpoint** Composite of CV death, nonfatal MI, or stroke: 6.6% in intervention group vs. 5.7% in control group (HR 1.167; 95% CI 0.742–1.835; *P =* 0.503) **Safety endpoint** TIMI major bleeding: 0.9% in intervention group vs. 0.7% in control group (*P =* 0.754)
		**Control arm**: Aspirin 100 mg od and Ticagrelor 90 mg bd	596				
ISAR-REACT 5, 2019 (18)	Randomized Open label Multicentre	**Intervention arm**: Aspirin 75 mg od and Ticagrelor 90 mg bd	2,012	12	UA: 12.7%; NSTEMI: 46.2%; STEMI: 41.1%	84.1%	**Efficacy endpoint** Composite of all-cause death, MI, or stroke: 9.3% in intervention group vs. 6.9% in control group (HR 1.36; 95% CI 1.09–1.70; *P =* 0.006) **Safety endpoint** BARC major bleeding: 5.4% in intervention group vs. 4.8% in control group (HR 1.12; 95% CI 0.83–1.51; *P =* 0.46)
		**Control arm**: Aspirin 75 mg od and Prasugrel 10 mg od	2,006				
TICAKOREA, 2019 (19)	Randomized Open label Multicentre	**Intervention arm**: Aspirin 100 mg od and Ticagrelor 90 mg bd **Control arm**: Aspirin 100 mg od and Clopidogrel 75 mg od	400: East Asian population: 400: East Asian population	12	UA: 21.3%; NSTEMI: 37.8%; STEMI: 40.7%	83.5%	**Efficacy endpoint** Composite of CV death, MI, stroke: 9.2% in intervention group vs 5.8% in control group (HR 1.62; 95% CI 0.96–2.74; *P =* 0.07) **Safety endpoint** Composite of major and minor bleeding according to PLATO criteria: 11.7% in intervention group vs. 5.3% in control group (HR 2.26; 95% CI 1.34–3.79; *P =* 0.002) Major bleeding was also higher in intervention group (*P =* 0.04)
POPular Genetics 2019 (20)	Randomized Open label Multicentre	**Intervention arm:** Aspirin plus P2Y_12_ inhibitor on the basis of early *CYP2C19* genetic testing (genotype-guided group)	1,242	12	STEMI: 100%	100%	**Primary endpoints** Net adverse clinical events (composite of death, MI, stent thrombosis, stroke, or PLATO major bleeding) 5.1% in intervention group vs. 5.9% in control group (absolute difference, −0.7; 95% CI −2.0 to 0.7; P < 0.001 for noninferiority) No significant difference in ischaemic endpoints. **Safety endpoint** PLATO major or minor bleeding (primary bleeding outcome) 9.8% in intervention group vs. 12.5% in control group (HR 0.78; 95% CI 0.61 to 0.98; *P =* 0.04).
		**Control arm:** Aspirin plus either ticagrelor or prasugrel	1,246				
POPular AGE, 2020 (21)	Randomized Open label Multicentre	**Intervention arm**: Aspirin 75 mg od and Clopidogrel 75 mg od	500: Elderly population	12	UA; 11%; NSTEMI: 86%	47%	**Efficacy endpoint** Composite of all-cause death, MI, stroke, or PLATO major and minor bleeding: 27% in intervention group vs. 32% in control group (absolute RR−4.3%; 95% CI −10·0 to 1·4; *P =* 0.025 for non-inferiority) No significant difference in ischaemic endpoints. **Safety endpoint** BARC major bleeding 3 & 5: 28% in intervention group vs. 46% in control group (HR 0.61; 95% CI 0·38–0·98; *P =* 0.034)
		**Control arm:** Aspirin 75 mg od and Ticagrelor 90 mg bd or Prasugrel 10 mg od	502: Elderly population				
HOST-REDUCE-POLYTECH-ACS, 2020 (22)	Randomized Open label Multicentre	**Intervention arm**: Aspirin 100 mg od and Prasugrel 10 mg od until 1 month, then Prasugrel reduced to 5 mg od for 11 months	1,170: East Asian population	12	UA: 60.8%; NSTEMI: 25.25%; STEMI: 13.95%	100%	**Primary Endpoints** Composite of all-cause death, nonfatal MI, stent thrombosis, repeat revascularization, stroke, and BARC ≥2 bleeding: 7.2% in intervention group vs. 10.1% in control group (HR 0.70; 95% CI 0.52–0.92, *P =* 0.012). No significant difference in ischaemic endpoints. **Safety Endpoint** BARC ≥3 bleeding: 0.8% in intervention group vs. 0.7% in control group (HR 1.12; 95% CI 0.43-2.90; *P =* 0.82)
		**Control arm:** Aspirin 100 mg od and Prasugrel 10 mg od for 12 months	1,168: East Asian population				
TALOS-AMI, 2021 (23)	Randomized Open label Multicentre	**Intervention arm**: Aspirin 100 mg od and Ticagrelor 90 mg bd for 1 month followed by 11 months Aspirin and Clopidogrel 75 mg od	1,349: East Asian population	12	NSTEMI: 46%; STEMI: 54%	100%	**Primary endpoints** Composite of CV death, MI, stroke, or BARC bleeding type 2, 3, or 5: 4.6% in intervention group vs. 8.2% in control group (HR 0·55; 95% CI 0.40–0.76; P noninferiority < 0.001, P superiority < 0.001) No significant difference in ischaemic endpoints **Safety endpoint** BARC 2,3, or 5 bleeding: 3.0% in intervention group vs. 5.6% in control group (HR 0.52; 95% CI 0.35–0.77; *P =* 0.001)
		**Control arm:** Aspirin 100 mg od and Ticagrelor 90 mg bd for 12 months	1,348: East Asian population				
GUARANTEE, (NCT03783351)	Randomized Open label Multicentre	**Intervention arm:** Genotyping done at 48 h following intervention. CYP2C19 *2 or *3 reduced function allele patients will receive Aspirin and Ticagrelor 90 mg bd, non-*2 or -*3 CYP2C19 patients will receive Clopidogrel 75 mg once daily	4,009: East Asian population	12	ACS and SA	100%	**Primary endpoints** Composite of all-cause death, non-fatal stroke, non-fatal MI and ischemia driven revascularization at one-year **Safety endpoint** Not specified
		**Control arm:** Patients will receive Aspirin with either Clopidogrel 75mg od or Ticagrelor 90mg bd, according to the clinical and procedural characteristics of patients					
**VERONICA, (NCT04654052)**	Randomized Open label Multicentre	**Intervention arm:** Aspirin and Ticagrelor or Prasugrel for 1 month, followed by platelet function testing. Patients with platelet reactivity units < 30, will de-escalate to Clopidogrel for 11 months	634	12	ACS	100%	**Primary Endpoints** Composite of CV death, stroke and all-cause death, non-fatal MI, or non-fatal stroke, and BARC type ≥ 2 bleeding **Safety Endpoint** BARC type ≥ 2 bleeding
		**Control arm:** Aspirin and Ticagrelor or Prasugrel for 1 month, followed by platelet function testing. Patients with platelet reactivity units < 30, will continue current treatment for 11 months					
ELECTA-SIRIO 2, (NCT04718025)	Randomized Open label Multicentre	**Intervention arm:** Aspirin 100 mg od and Ticagrelor 90 mg BD for 1 month, followed by Aspirin 100 mg od and Ticagrelor 60 mg bd for 11 months Or Aspirin 100 mg od and Ticagrelor 90 mg bd for 1 month, followed by Ticagrelor 60 mg monotherapy for 11 months	4,500	12	ACS	100%	**Primary endpoint** Composite of death from any cause, MI or non-fatal stroke **Secondary endpoint** BARC 2,3 or 5 major bleeding.
		**Control arm:** Aspirin 100 mg od and Ticagrelor 90 mg bd for 12 months					

Studies are listed in chronological order of publication date. Those enrolling a particular selected population such as East Asian or elderly patients, are indicated.

ACS, acute coronary syndromes; BARC, Bleeding Academic Research Consortium; CABG, coronary artery bypass grafting surgery; CI, confidence interval; CV, cardiovascular; DAPT, dual antiplatelet therapy; HR, hazard ratio; MI, myocardial infarction; NSTEMI, non-ST-elevation myocardial infarction; PCI, percutaneous coronary intervention; PLATO, PLATelet inhibition and patient Outcomes; RR, risk reduction; SA, stable angina; STEMI, ST-elevation myocardial infarction; TIMI, Thrombolysis in myocardial infarction; UA, unstable angina.

**Table 2:** De-escalation of dual antiplatelet therapy duration in ACS population undergoing PCI.

**Study, year**	**Study design**	**DAPT strategy**	**Population (n)**	**Follow up (months)**	**ACS (%)**	**PCI (%)**	**Results**
EXCELLENT, 2011 (24)	Randomized Open label Multicentre	**Intervention arm:** Aspirin 100–200 mg plus Clopidogrel 75 mg for 6 months and thereafter Aspirin alone	722: East Asian population	12	UA/NSTEMI: 48%; STEMI:3%	100%	**Efficacy endpoint** Composite of cardiac death, MI, or ischaemia–driven target vessel revascularization: 4.8% in intervention group vs, 4.3% in control group (95% CI, 2.4%; *P =* 0.001 for noninferiority) **Safety endpoint** TIMI major bleeding: 0.3% in intervention group vs. 0.6% in control group (HR 0.50; 95% CI 0.09–2.73, *P =* 0.42)
		**Control arm:** Aspirin 100–200 mg plus Clopidogrel 75 mg for 12 months	721: East Asian population				
I-LOVE-IT 2 2016 (25)	Randomized Single-blinded Multicentre	**Intervention arm**: DAPT (Aspirin plus P2Y_12_ inhibitor) for 6 months, followed by Aspirin alone	909: East Asian population	12	STEMI: 14%; NSTEMI: 11%; Asymptomatic: 4%	100%	**Efficacy endpoint** Target lesion failure (composite of cardiac death, target vessel MI or target lesion revascularization): 6.8% in intervention group vs. 5.9% in control group (absolute difference 0.87%; 95% CI −1.37% to 3.11%, P noninferiority = 0.0065) **Safety endpoint** NACE and cerebral events (composite of all–cause death, MI, stroke, or major BARC type ≥3 bleeding): 7.8% in intervention group vs. 7.3% in control group (*P =* 0.6)
		**Control arm**: DAPT (Aspirin plus P2Y_12_ inhibitor) for 12 months	920: East Asian population				
SMART-DATE, 2018 (26)	Randomized Open label Multicentre	**Intervention arm**: Aspirin 100 mg od plus a P2Y_12_ inhibitor (Clopidogrel/ Ticagrelor/ Prasugrel) for 6 months and thereafter Aspirin alone	1,357: East Asian population	18	UA; 31.0%; NSTEMI 31.5%; STEMI; 37.5%	100%	**Efficacy endpoints** Composite of all–cause death, MI, or stroke: 4.7% in intervention group vs. 4.2% control group (HR 1.13; 95% CI 0.79–1.62; *P =* 0.51) MI occurred more frequently in the 6–month DAPT group than in the 12–month or longer DAPT group (1.8% vs. 0.8%; HR 2·41; 95% CI 1.15–5.05; *P =* 0.02) **Safety endpoint** BARC type 2–5 bleeding: 2.7% in intervention group vs. 3.9% in control group (HR 0.59; 95% CI 0.45–1.05; *P =* 0.09)
		**Control arm**: Aspirin 100 mg od plus a P2Y_12_ inhibitor (Clopidogrel/ Ticagrelor/ Prasugrel) for at least 12 months	1,355: East Asian population				
GLOBAL LEADERS, 2018; (ACS Subgroup) (27, 28)	Randomized Open label Multicentre	**Intervention arm**: Aspirin 75–100 mg od and Ticagrelor 90 mg bd for 1 month, followed by 23 months of Ticagrelor	3,750	24	UA: 27%; NSTEMI: 45%; STEMI: 28%	99.6%	**Efficacy endpoint** Composite of all-cause mortality or nonfatal MI: 3.92% in intervention group vs. 4.52% in control group (RR 0.86; 95% CI 0.69–1.08; *P =* 0.189) **Safety endpoint** Site-reported BARC grade 3 or 5 bleeding: 1.95% in intervention group vs. 2.68% in control group (RR 0.73; 95% CI 0.54–0.98; *P =* 0.037)
		**Control arm**: Aspirin 75−100 mg od and Ticagrelor 90 mg bd for 12 months, followed by 12 months Aspirin monotherapy	3,737				
REDUCE 2019 (29)	Randomized Open label Multicentre	**Intervention arm**: DAPT (Aspirin plus P2Y_12_ inhibitor) for 3 months, followed by Aspirin alone	751	24	STEMI: 47%; NSTEMI: 38%; UA: 15%	100%	**Efficacy endpoint** Composite outcome of composite of all–cause death, MI, stent thrombosis, stroke, target vessel revascularisation and bleeding: 8.2% in intervention group vs. 8.4% in control group (P non–inferiority < 0.001) No significant difference in ischaemic endpoints. **Safety endpoint** BARC 2, 3 or 5 bleeding: 3.3% in intervention group vs. 4.0% in control group (*P =* 0.46)
		**Control arm**: DAPT (Aspirin plus P2Y_12_ inhibitor) for 12 months	745				
TWILIGHT, 2019 (30)	Randomized Double-Blinded Multicentre	**Intervention arm**: Aspirin 81–100 mg and ticagrelor 90 mg bd for 3 months followed by Ticagrelor and placebo for further 12 months	3,555	15	No–symptoms: 6.45%; SA: 28.75%; UA: 35%; NSTEMI: 29.8%	100%	**Efficacy endpoint** Composite outcome of all–cause death, MI, or stroke: 3.9% in both groups (HR 0.99; 95% CI 0.78–1.25; P non–inferiority < 0.001) **Safety endpoint** BARC 2, 3 or 5 bleeding: 4.0% in intervention group vs. 7.1% in control group (HR 0.56; 95% CI 0.45–0.68, *P* < 0.001).
		**Control arm**: Aspirin 81–100 mg od and Ticagrelor 90 mg bd for 15 months	3,564				
SMART-CHOICE, 2019 (31)	Randomized Open label Multicentre	**Intervention arm**: Aspirin 75 mg od and a P2Y_12_ inhibitor (Clopidogrel/ Ticagrelor/ Prasugrel) for 3 months followed by a P2Y_12_ inhibitor for 9 months	1,495: East Asian population	12	SA: 41.8%; UA: 32%; NSTEMI: 15.7%; STEMI: 10.5%	100%	**Efficacy endpoint** Composite of all–cause death, MI, or stroke: 2.9% in intervention group vs. 2.5% in control group (Absolute difference 0.4%; 95% CI –∞% to 1.3%; P noninferiority = 0.007; P superiority = 0.46) **Safety endpoint** BARC 2–5 Bleeding: 2.0% in intervention group vs. 3.4% in control group (HR 0.58; 95% CI 0.36–0.92; *P =* 0 0.02)
		**Control arm**: Aspirin 75 mg od and a P2Y_12_ inhibitor (Clopidogrel/ Ticagrelor/ Prasugrel) for 12 months	1,498: East Asian population				
STOPDAPT-2, 2019 (32)	Randomized Open label Multicentre	**Intervention arm**: 1 month Aspirin 81–200 mg and either Clopidogrel 75 mg od or Prasugrel 3.75 mg od at physician's discretion. At 1 month, Aspirin stopped and Clopidogrel monotherapy continued	1,500: East Asian population	12	SA: 62%; UA: 13.5%; NSTEMI: 6%; STEMI: 18.7%	100%	**Primary endpoints** Composite of CV death, MI, stroke, stent thrombosis, or TIMI major or minor bleeding: 2.36% in intervention group vs. 3.70% in control group (HR 0.64; 95% CI 0.42–0.98; meeting criteria for noninferiority P < 0.001 and for superiority *P =* 0 0.04) No significant difference in ischaemic endpoints. **Safety endpoint** TIMI major/ minor bleeding: 0.41% in intervention group vs. 1.54% in control group (HR 0.26; 95% CI 0.11–0.64; *P =* 0.004 for superiority) BARC 3 or 5 Bleeding: 0.54% in intervention group vs. 1.81% in control group (HR 0.30; 95% CI 0.13–0.65; *P =* 0 0.003)
		**Control arm**: Aspirin 81–200 mg and either Clopidogrel 75 mg or Prasugrel 3.75 mg od for up to 12 months. Patients on Prasugrel switched to Clopidogrel at 1 month in both groups for a further 11 months	1,509: East Asian population				
STOPDAPT-2 ACS, 2019 (33)	Randomized Open label Multicentre	**Intervention arm**: 1–2 months Aspirin 81–200 mg and either Clopidogrel 75 mg od or Prasugrel 3.75 mg od at physician's discretion. At 1 month, Aspirin stopped and Clopidogrel continued	2,058: East Asian population	12	UA; 57%; NSTEMI; 20%; STEMI; 24%	100%	**Primary endpoints** Composite of CV death, MI, stroke, definite stent thrombosis, or TIMI major or minor bleeding: 3.2% in intervention group vs. 2.8% in control group (HR 1.14, 95% CI 0.80–1.62, P for noninferiority =0.06 and for superiority P not significant) Numerical increase noted in MI events. **Safety endpoint** TIMI major/ minor bleeding: 0.5% in intervention group vs. 1.2% in control group (HR 0.46; 95% CI 0.23–0.94) BARC 3 or 5 Bleeding: 0.5% in intervention group vs. 1.3% in control group (HR 0.41; 95% CI 0.20–0.83)
		**Control arm**: Aspirin 81–200 mg and either Clopidogrel 75 mg or Prasugrel 3.75 mg od for up to 12 months. Patients on Prasugrel switched to Clopidogrel at 1 month in both groups for a further 11 months	2,057: East Asian population				
TICO, 2020 (34)	Randomized Open label Multicentre	**Intervention arm**: Aspirin 100 mg od and Ticagrelor 90 mg bd for 3 months followed by Ticagrelor monotherapy for 9 months thereafter	1,527: East Asian population	12	UA: 30.5%; NSTEMI: 33.5 %; STEMI: 36%	100%	**Primary endpoints** Composite of death, MI, stent thrombosis, stroke, target vessel revascularization, and TIMI major bleeding: 3.9% in intervention group vs. 5.9% in control group (HR 0.66; 95% CI 0.48–0.92; *P =* 0.01) No significant difference in ischaemic endpoints. **Safety endpoint** Major bleeding (TIMI criteria): 1.7% in intervention group vs. 3.0% in control group (HR 0.56; 95% CI 0.34–0.91, *P =* 0.02)
		**Control arm**: Aspirin 100 mg od and Ticagrelor 90 mg bd for 12 months	1,529: East Asian population				
MASTER DAPT, 2021 (35)	Randomized Open label Multicentre	**Intervention arm**: 1–month DAPT with Aspirin and either Ticagrelor, Clopidogrel or Prasugrel, followed by monotherapy with either Aspirin or Ticagrelor, Prasugrel or Clopidogrel at physician's discretion	2,295	12	NSTEMI: 26%; STEMI: 12%; Silent; Ischaemia: 11%	100%	**Primary endpoints** Composite of all–cause mortality, MI, stroke, or major bleeding BARC 3 or 5: 7.5% in intervention group vs. 7.7% in control group (HR 0.97; 95% CI 0.78–1.20; *P* < 0.001 for noninferiority) No significant difference in ischaemic endpoints. **Safety endpoint** Major or clinically relevant nonmajor bleeding BARC type 2, 3, or 5: 6.5% in intervention group vs. 9.4% in control group (HR 0.64; 95% CI 0.55–0.85; *P* < 0.001 for superiority)
		**Control arm**: DAPT with Aspirin and either Ticagrelor, Clopidogrel or Prasugrel for 3–12 months, followed by monotherapy with either Aspirin or Ticagrelor, Prasugrel or Clopidogrel at physician's discretion	2,284				
DUAL-ACS2, (NCT03252249)	Randomized Open label Multicentre	**Intervention arm:** 3 months of DAPT	18,318	15	ACS	100%	**Primary endpoint** All–cause death **Safety endpoints** Major fatal and non–fatal bleeding
		**Control arm:** 12 months of DAPT					
Target DAPT, (NCT03008083)	Randomized Open label Multicentre	**Intervention arm:** Aspirin and either Ticagrelor 90 mg bd or Clopidogrel 75 mg od for 3 months, followed by Aspirin monotherapy.	2,446: East Asian Population	36	SA	100%	**Primary endpoint** Composite of all–cause death, MI, stroke, and major bleeding at 18 months **Safety endpoint** BARC major bleeding Gusto major bleeding
		**Control arm:** DAPT with P2Y_12_ inhibitors and Aspirin up to 360 days, after which patients will continue on monotherapy with Aspirin only					
IVUS ACS and Ultimate DAPT Trials, (NCT03971500)	Randomized Open label Multicentre	**Intervention arm**: IVUS guided PCI. Aspirin and Ticagrelor for 1 month and a further randomization to either 11 months of Aspirin and Ticagrelor or Ticagrelor alone	3,486: East Asian Population	12	ACS	100%	**Primary endpoint** Target vessel failure at 12 months between angiography and IVUS guided PCI groups. Major adverse cardiovascular and stroke at 1 month from randomization to single antiplatelet or DAPT. **Safety endpoint** BARC≥2 bleeding at 1 month of randomization to single antiplatelet therapy or DAPT. .
		**Control arm**: Angiography guided PCI. Aspirin and Ticagrelor for 1 month and a further randomization to either 11 months of Aspirin and Ticagrelor or Ticagrelor alone					
NEOMINDSET (NCT04360720)	Randomized Open label Multicentre	**Intervention arm:** Ticagrelor 90 mg bd or Prasugrel 10 mg od after randomization. Aspirin discontinued immediately after randomization	3,400	12	ACS	100%	**Primary endpoint** Composite endpoint of all–cause death, stroke, MI, or urgent target vessel revascularization **Safety endpoint** BARC 2, 3 or 5 bleeding
		**Control arm:** Aspirin 100 mg od and either Ticagrelor 90 mg bd or Prasugrel 10 mg od					
STOPDAPT-3, (NCT04609111)	Randomized Open label Multicentre	**Intervention arm:** Prasugrel 10 mg monotherapy before index PCI procedure to one month followed by Clopidogrel monotherapy for 11 months	3110: East Asian Population	12	ACS and SA	100%	**Primary endpoint** Composite of CV death, MI, ischemic stroke, or definite stent thrombosis **Safety endpoint** BARC 3 or 5 bleeding
		**Control arm:** Aspirin with Prasugrel 10 mg od for 1 month followed by Aspirin monotherapy					
CAGEFREE II, (NCT04971356)	Randomized Open label Multicentre	**Intervention arm:** Aspirin 100 mg od and Ticagrelor 90 mg bd for one month, followed by Ticagrelor 90 mg bd for 5 months, and Aspirin 100 mg od for 6 months thereafter	1908: East Asian Population	12	ACS	100%	**Primary endpoint** Composite of all–cause death, stroke, MI, any revascularization, and BARC type 3 or 5 bleeding **Safety endpoint** BARC type 3 or 5 bleeding
		**Control arm:** Aspirin 100 mg od and Ticagrelor 90 mg bd for 12 months					
LEGACY, (NCT05125276)	Randomized Open label Multicentre	**Intervention arm:** Clopidogrel, Ticagrelor or Prasugrel only for 12 months	3090	12	ACS	100%	**Primary endpoint** Composite of all–cause mortality, MI, and stroke **Safety endpoint** BARC 2,3, or 5 bleeding
		**Control arm:** Aspirin 75–100 mg and either Clopidogrel, Ticagrelor or Prasugrel for 12 months					
BULK-STEMI, (NCT04570345)	Randomized Open label Multicentre	**Intervention arm:** 3 months of Aspirin and Ticagrelor followed by Ticagrelor monotherapy for 9 months	1,002: East Asian Population	12	STEMI; 100%	100%	**Primary endpoint** Composite of all–cause mortality, MI, stroke, stent thrombosis and BARC major bleeding **Safety endpoint** BARC 3, 5 major bleeding
		**Control arm:** Aspirin and Ticagrelor for 12 months					
Optimized-APT, (NCT04338919)	Randomized Open label Multicentre	**Intervention arm:** Aspirin 75 mg od and Ticagrelor 90 mg bd for the first month, followed by ticagrelor 90 mg monotherapy months 2–6 and ticagrelor 45 mg bd monotherapy from months 7–12	2,020: East Asian population	12	ACS	100%	**Primary endpoint** Composite of death from CV causes, non–fatal MI, stent thrombosis, ischemia driven coronary revascularization and ischaemic stroke. **Secondary endpoint** Plato major bleeding BARC 2, 3 or 5 major bleeding.
		**Control arm:** Aspirin 75 mg od and Ticagrelor 90 mg bd for 12 months					

Studies are listed in chronological order of publication date. Those enrolling a particular selected population such as East Asian or elderly patients, are indicated.

ACS, acute coronary syndromes; BARC, Bleeding Academic Research Consortium; CABG, coronary artery bypass grafting surgery; CI, confidence interval; CV, cardiovascular; DAPT, dual antiplatelet therapy; HR, hazard ratio; MI, myocardial infarction; NSTEMI, non-ST-elevation myocardial infarction; PCI, percutaneous coronary intervention; RR, risk reduction; SA, stable angina; STEMI, ST-elevation myocardial infarction; TIMI, Thrombolysis in myocardial infarction; UA, unstable angina; IVUS, intravascular ultrasound.

## Reduced intensity DAPT or de-escalation of DAPT intensity

Trials assessing the safety and efficacy of various de-escalation strategies performed a head-to-head comparison of (i) more potent DAPT, containing ticagrelor or prasugrel, with DAPT containing clopidogrel, or (ii) potent DAPT for 6–12 months with potent DAPT only for 1–4 weeks followed by de-escalation to clopidogrel or low dose prasugrel, or (iii) DAPT containing prasugrel to DAPT containing ticagrelor (Table 1) (10–23). We highlight some idiosyncrasies below and indicate which category above (i–iii) the study belongs to.

The single-center TOPIC trial (ii) showed that de-escalation of DAPT intensity at 1 month post-ACS from aspirin plus ticagrelor or prasugrel to aspirin plus clopidogrel, was superior to 12 months of aspirin plus ticagrelor or prasugrel, with a reduction in the composite of ischaemic and bleeding endpoints, driven by a reduction in major bleeding (15). Notably, the primary endpoint of the composite of cardiovascular death, unplanned hospitalization leading to urgent coronary revascularization, stroke, and bleeding academic research consortium (BARC) ≥2 bleeding, did not specifically include MI, although most likely would have been captured by unplanned hospitalization.

De-escalation guided by platelet function testing (PFT) was assessed in the TROPICAL-ACS study (ii) (14). Here, DAPT comprising of aspirin plus prasugrel was compared with de-escalation to clopidogrel. In the de-escalation arm, prasugrel was given for 1 week, followed by clopidogrel for 1 week, then PFT was conducted using the Multiplate Analyzer. If high platelet reactivity was documented, patients were switched back to prasugrel, otherwise clopidogrel was continued. The primary endpoint of the composite of cardiovascular death, MI, stroke, or bleeding (BARC ≥ 2) occurred less often in the guided de-escalation group than in the control group, with no significant difference in ischaemic endpoints or BARC ≥2 bleeding, but a reduction in the secondary endpoint of BARC 3 or 5 bleeding (14).

The PRASFIT-ACS study (i) compared DAPT comprising of low dose prasugrel (3.75 mg daily) plus aspirin to DAPT containing clopidogrel plus aspirin (10). The primary endpoint of MACE at 24 weeks occurred in 9.4% of the prasugrel and 11.8% of the clopidogrel group, showing use of lower dose prasugrel (3.75 mg) in East Asians seems to achieve similar effects to those seen in TRITON-TIMI 38 with full-dose prasugrel compared to clopidogrel in predominantly Western patients (7).

The HOST-REDUCE-POLYTECH-ACS trial (ii) evaluated de-escalation of DAPT at 1-month post-ACS, from 10 to 5 mg prasugrel, in combination with aspirin for 12 months, in Korea (22). Standard-dose prasugrel 10 mg daily was associated with higher bleeding rates than the same dose in Western populations (39, 40). Interestingly, a subsequent pre-specified subgroup analysis showed that whilst prasugrel de-escalation decreased NACE due to a reduction in bleeding, this benefit was confined to non-ST segment elevation ACS (NSTE-ACS) patients and not seen in patients with STEMI (41).

The POPular Genetics study (i) assessed the use of lower intensity DAPT, guided by *CYP2C1* genotyping, against standard DAPT containing ticagrelor or prasugrel, in patients undergoing primary PCI (20). In the genotype-guided group, carriers of *CYP*2*C*19^*^2 or *CYP*2*C*19^*^3 loss-of-function alleles received ticagrelor or prasugrel (39%), and noncarriers received clopidogrel (61%). Genotype-guided use of reduced intensity DAPT was noninferior to standard DAPT with respect to thrombotic events and significantly reduced bleeding.

In the POPular AGE trial (i), patients with NSTE-ACS aged 70 or more years were randomized to DAPT comprising of either aspirin plus clopidogrel or aspirin plus prasugrel or ticagrelor (21). In the control arm, 93.8% of patients received ticagrelor. Aspirin plus clopidogrel met the criteria for non-inferiority with respect to NACE and for superiority with respect to PLATO major and minor bleeding. Importantly, since only 47% of patients underwent PCI, the study was under-powered to assess the safety of de-escalation in this cohort with respect to ischaemic endpoints.

The Elderly-ACS 2 trial (i) in patients aged >74 years with ACS undergoing PCI compared DAPT comprising of aspirin plus low-dose prasugrel (5 mg daily) to aspirin plus clopidogrel for 12 months (16). The study was terminated prematurely for futility following a planned interim analysis. There was no difference in the primary endpoint of all-cause death, MI, stroke, rehospitalization or bleeding, or the secondary endpoint of BARC ≥2 bleeding, although stent thrombosis occurred more frequently in patients taking clopidogrel compared to those taking prasugrel.

## De-escalation of DAPT duration

Eleven studies assessed de-escalation of DAPT duration from 12 months to a shorter period (Table 2) (24–35). Some of the earliest studies had relatively small sample size, with lower than expected rates of adverse events (29). The GLOBAL LEADERS trial in patients undergoing PCI for stable coronary disease or ACS, compared aspirin plus ticagrelor for 1 month, followed by 23 months of ticagrelor monotherapy, or standard DAPT with aspirin daily plus either clopidogrel (for patients with stable coronary disease) or ticagrelor (for patients with ACS) for 12 months, followed by aspirin monotherapy for 12 months (27). The trial failed to show any benefit at 2 years on the primary endpoint of the composite of all-cause death and MI. However, abbreviated DAPT reduced bleeding in the ACS subgroup (28).

The TWILIGHT study evaluated de-escalation of DAPT from aspirin and ticagrelor, to ticagrelor alone, at 3 months post-PCI, with 65% of patients undergoing PCI (30). De-escalation reduced the incidence of clinically-relevant bleeding, without an increase in death, MI or stroke.

The MASTER DAPT study compared short-term DAPT (1 month) followed by monotherapy with clopidogrel (54%) or aspirin, with DAPT for 3 months or more, in post-PCI patients at high bleeding risk, and 40% of patients had an ACS presentation (35). Whilst the results showed that 1-month was noninferior to 3 months or more DAPT for NACE, and superior for reducing the composite of major or clinically relevant nonmajor bleeding, it should be noted that the latter included BARC 2 as well as BARC 3 and 5 bleeding and that 37% of patients were receiving anticoagulation.

The STOPDAPT-2 was an open label randomized trial in patients with ACS (38%) or stable angina, randomized to either 1 month of DAPT followed by clopidogrel monotherapy or to 12 months of DAPT with aspirin and clopidogrel (32). Abbreviated DAPT met the criteria for noninferiority and superiority compared with 12-months DAPT for the composite primary endpoint of cardiovascular death, MI, stroke, stent thrombosis, or major or minor bleeding, including in ACS patients. However, in the subsequent STOPDAPT-2 ACS trial in patients with ACS undergoing PCI, 1-month DAPT followed by clopidogrel monotherapy did not meet the criteria for non-inferiority compared to 12 months of DAPT with respect to NACE, comprising of cardiovascular death, MI, stroke, stent thrombosis or bleeding (including minor bleeding). There was a trend toward harm with a 2-fold increase in MI with the 1-month DAPT regimen, although there was a reduction in bleeding (33).

The SMART-DATE trial compared 6 months of DAPT followed by aspirin alone to conventional 12 months DAPT (26). Although there was no difference in the composite of all-cause death, MI, or stroke, with 6 months DAPT meeting criteria for non-inferiority, there was a significantly increase in MI with 6 vs. 12 months of DAPT, without a reduction in bleeding.

The SMART-CHOICE trial randomized patients receiving PCI to either continue or to stop aspirin after 3 months of DAPT. Around 58% of patients had ACS and some 77% of patients had clopidogrel as the P2Y_12_ inhibitor in combination with aspirin (31). The composite of all-cause death, MI, or stroke at 12 months was similar between the study arms, with a reduction in bleeding with abbreviated DAPT.

## Discussion

The TRITON-TIMI 38 and PLATO trials showed that the greatest ischaemic benefit from DAPT with a P2Y_12_ inhibitor was achieved early, within the first 30 days post-ACS, and that the bleeding risk was mainly apparent beyond this (7, 9). A number of trials subsequently assessed de-escalation of DAPT either through reduction in DAPT intensity or duration.

Overall, de-escalation of DAPT duration post-ACS to monotherapy appears favorable, with reduction in bleeding, mostly without increase in MACE, although an increase in ischaemic events was noted in some studies with abbreviated DAPT. Likewise, de-escalation of DAPT intensity appears to significantly reduce major bleeding, without significant effect on MACE. Importantly, these approaches have not been tested with adequately powered trials in patients at high ischaemic risk, therefore these approaches should be generally confined to low ischaemic, high bleeding risk patients.

Importantly, most of the studies showing a benefit of de-escalation of DAPT intensity were conducted in East Asian patients, who are more prone to bleeding (39). In Westerners, the strategy of de-escalation of DAPT intensity from ticagrelor or prasugrel to clopidogrel, after a short period of more intense DAPT, was only evaluated in two relatively small studies, one of which used PFT to guide de-escalation (14, 15). Combining all studies, in East Asian, Western and elderly patients, the use of lower intensity P2Y_12_ inhibitor, namely clopidogrel, compared to ticagrelor or prasugrel, appears to have no significant impact on net adverse events, although it is important to look at different populations where specific bleeding or ischaemic risks may predominate. Specifically, comparing the efficacy of clopidogrel to ticagrelor or prasugrel as part of DAPT, the evidence, largely driven by the original PLATO and TRITON-TIMI 38 studies, indicates a trend toward increased MACE and reduction of major bleeding with clopidogrel. The reduction in major bleeding in TOPIC and TROPICAL-ACS had very wide confidence intervals and one of the studies used a guided-de-escalation with PFT, and whilst the POPular GENETICS study showed reduced bleeding, the evidence cannot confidently support this approach in the broad population, especially without genetic or PFT testing to guide treatment. In East Asian patients with relatively low thrombogenic milieu, (42) de-escalation of DAPT intensity from appears to have no significant effect on ischaemic endpoints, but significantly reduces major bleeding. On the other hand, whilst most studies in East Asian patients have shown that reduction of DAPT duration significantly reduces NACE and bleeding, there are two studies, SMART-DATE and STOPDAPT-2 ACS, which indicate a possible increase in ischaemic risk with reduced DAPT duration. A similar signal was seen in the subgroup analysis of the HOST-REDUCE-POLYTECH-ACS study (22). However, some studies in East Asian patients used prasugrel 3.75 mg daily (10, 32, 33), a dose that has not been tested for efficacy in Western patients. Furthermore, the type and potency of antiplatelet agent used as monotherapy can be related to an increased risk of thrombotic events during the early phase of ACS. In the elderly, lower intensity DAPT appears to reduce bleeding, without increasing ischaemic events.

A recent network meta-analysis compared the two de-escalation strategies in ACS patients undergoing PCI, namely shorter DAPT vs. de-escalation of DAPT intensity (43). Whilst there was no difference in all-cause mortality, de-escalation overall reduced NACE (trial defined composite of MI, stroke, stent thrombosis, and minor bleeding), while shortened DAPT decreased major bleeding. Another meta-analysis of 19 randomized controlled trials assessing de-escalation of DAPT in ACS concluded that compared to personalized de-escalation guided by PFT or genotyping, unguided de-escalation was as safe, if not safer, with decreased bleeding and without excess ischemic risk (44). Notably that meta-analysis included patients not receiving PCI, and guided de-escalation was predominantly assessed in Westerners, whereas unguided de-escalation predominantly in East Asians. Another meta-analysis of guided vs. standard DAPT in patients undergoing PCI, showed that guided de-escalation reduced MACE, including its components, with reduction in minor but not major bleeding (45). However, that metanalysis included 11 randomized and 3 observational studies utilizing both escalation and de-escalation of antiplatelet therapy, included patients with chronic coronary syndrome, and some studies used non-conventional antiplatelet therapy namely cilostazol or double-dose clopidogrel. Whilst there has been no head-to-head comparison of genotyping or PFT guided de-escalation, subgroup analysis showed no difference in outcomes whether PFT or genotyping was utilized to guide DAPT (45). Indeed, there are pros and cons to both strategies, which is beyond the scope of this review, and a combined approach using both strategies may have added advantages, but has not been evaluated.

## Limitations of the current review

Our review has a number of potential limitations. Firstly, there is heterogeneity in reporting bleeding, with various definitions used including BARC, PLATO and TIMI classifications. Even amongst studies that included the same classification of bleeding (e.g., BARC), some studies have included BARC 2, 3 and 5 bleeding events, whilst others included only BARC 3 and 5. There was also heterogeneity in the populations studied, with some only assessing ACS patients undergoing PCI, whilst others included patients with chronic coronary syndrome or some medically-managed ACS patients. The regimens and doses of antiplatelet agents varied, particularly in studies conducted in East Asia, where lower doses of prasugrel were used. There was heterogeneity amongst studies with respect to the monotherapy (SAPT) continued after shortened DAPT, some continuing with aspirin, whilst others continuing ticagrelor or clopidogrel. The duration of “shortened” DAPT also varied from 1 to 6 months. Amongst the studies investigating de-escalation of DAPT intensity, there was heterogeneity in the “intense” regimen with some studies giving ticagrelor, some prasugrel and some either prasugrel or ticagrelor. Many studies were open label and generally, high risk bleeding patients were underrepresented. Some studies included patients taking oral anticoagulation.

## Current research gaps

There are currently a number of gaps, which limit the applicability of these trial results to the main population of patients with ACS undergoing PCI.

There has been no direct head-to-head comparison of de-escalation of DAPT intensity with de-escalation of DAPT duration, and this is a significant limitation for the clinician, when attempting to choose an option to reduce bleeding risk.

Whilst it would appear sensible to de-escalate either DAPT intensity or duration in high bleeding risk patients, in practice it is difficult to separate patients at high bleeding risk, from those at high ischaemic risk, with overlapping risk factors including age and renal impairment.

Furthermore, no trial has assessed de-escalation strategies in high ischaemic risk patients, namely those with ST-elevation MI with multiple or extensive stenting, patients with residual disease, renal impairment, or severe left ventricular impairment. Lastly, several studies also included non-ACS patients, and those were generally under-powered to assess outcomes purely in the ACS subgroups.

## Potential future directions

Whilst a number of studies are ongoing (Tables 1, 2), there is a need to assess a combined approach, namely de-escalation of both intensity and duration, together, in patients at high bleeding risk, particularly the elderly. Furthermore, following abbreviated DAPT, the different drug options for SAPT, namely aspirin, clopidogrel or ticagrelor, need to be compared, to identify the optimal monotherapy, either empirically or guided by PFT.

Another gap in evidence is classifying patients in a uniformly applicable way, to high bleeding risk, high ischaemic risk, or both. This would enable clinicians to apply the results of such trials more easily to everyday practice.

Incorporation of risk scores or biomarkers of ischaemic or bleeding risk, such as high-sensitivity C-reactive protein and platelet function, into future trials would help identify patients who may benefit from and who may potentially come to harm, with de-escalation.

There have been no trials assessing shorter DAPT duration in the elderly. With an aging population and bleeding complications occurring typically 1–12 months post-ACS, this is an unmet need. Women are generally at higher bleeding risk than men with DAPT, yet women form only a minority of patients in most studies. High platelet reactivity significantly increases the risk of thrombosis only in men, whereas this phenotype is mainly associated with reduced bleeding only in women (46). Thus, specific trials in women, or patient-level data analyses combining the results of trials to date would be useful to identify optimal DAPT intensity or duration in women.

## Author contributions

All authors have made significant contributions to the manuscript that justifies authorship, read, and approved the final manuscript.

## Conflict of interest

The authors declare that the research was conducted in the absence of any commercial or financial relationships that could be construed as a potential conflict of interest.

## Publisher's note

All claims expressed in this article are solely those of the authors and do not necessarily represent those of their affiliated organizations, or those of the publisher, the editors and the reviewers. Any product that may be evaluated in this article, or claim that may be made by its manufacturer, is not guaranteed or endorsed by the publisher.

## Abbreviations

ACS: acute coronary syndrome
BARC: bleeding academic research consortium
DAPT: dual antiplatelet therapy
MACE: major adverse cardiovascular events
NACE: net adverse cardiovascular events
MI: myocardial infarction
PCI: percutaneous coronary intervention
PFT: platelet function testing
SAPT: single antiplatelet therapy.
